# Effect of porous material and black coating on solar desalination for sustainable water harvesting: A thermo-exergo-economic and environmental analysis

**DOI:** 10.1038/s41598-025-31246-0

**Published:** 2025-12-29

**Authors:** Naveen Sharma, Ajay Kumar, Robert Cep, Monika, Ajit Katiyar

**Affiliations:** 1 Department of Mechanical Engineering, Netaji Subhas University of Technology, New Delh, 110078 India; 2 Department of Mechanical Engineering, School of Engineering, Faculty of Science, Technology & Architecture, Manipal University Jaipur, Jaipur, 303007 Rajasthan India; 3 Department of Machining, Assembly and Engineering Metrology, Faculty of Mechanical Engineering, VSB-Technical University of Ostrava, 70800 Ostrava, Czech Republic; 4 Department of Geoinformatics, Netaji Subhas University of Technology, New Delh, 110078 India; 5 Department of Mechanical Engineering, Manav Rachna University, Faridabad, Haryana 121004 India

**Keywords:** Black coating, Porous material, Productivity enhancement, Solar energy, Thermo-economic analysis, Energy science and technology, Engineering, Environmental sciences

## Abstract

The aim of this research is to improve the amount of freshwater yield of a square base pyramidal solar still (SBPSS) using porous material with and without black coating for sustainable water harvesting. Seven separate cases, i.e., smooth basin (C-A1), an absorber plate with clay pots facing upward (C-A2), upward-downward (C-A3), downward (C-A4) and clay pots with black coating facing upward (C-A5), upward-downward (C-A6), downward (C-A7), were tested under the meteorological conditions of Andhra Pradesh (16.6834°N, 80.3904°E), India. To mitigate the impact of fluctuations in solar radiation, the tests were carried out specifically from 7:00 AM to 7:00 PM, focusing on days, especially, with clear sky. The influence of porous material and black coating on the performance of SBPSS is investigated from the viewpoints of thermo-exergo-economic parameters. By putting clay pots inside the basin, the freshwater yield increases up to 2.04 L/m^2^ for C-A4, while the yield enhances further by black coating (C-A7) to 2.38 L/m^2^. Findings revealed the overall daily thermal efficiencies for C-A7, C-A6, C-A4, C-A5, C-A3, C-A2, and C-A1 as 32.17%, 30.26%, 28.10%, 27.74%, 26.49%, 25.41%, and 17.00%, respectively. Additionally, the daily exergy values are 1.25%, 1.38%, 1.52%, 1.70%, 1.86%, 2.23%, and 2.78% for C-A1, C-A2, C-A3, C-A4, C-A5, C-A6, and C-A7. The optimal configuration was C-A7, exhibiting thermal and exergy efficiencies that surpassed C-A1 by 89.24% and 122.58%, respectively. Moreover, the daily productivity improved by 90.76%, accompanied by a cost reduction of 44.51%, and a reduction in payback period by 159 days. Among all, C-A5 achieves the highest CO_2_ mitigation at 7.06 tons/year, followed by C-A7 at 6.63 tons/year.

## Introduction

Enhanced urban growth, shifts in climate patterns, and pollution of groundwater sources are contributing to a scarcity of fresh water. In the span of a century, the demand for water has surged by approximately 600%. An estimated 7.7 billion people worldwide are grappling with limited access to water. The global water requirement stands at roughly 4600 km^3^ per year and is projected to rise by 20–30% by the year 2050^1^. Further, the contamination of potable water sources by infectious agents, organic waste, fertilizers, heavy metals, and emerging pollutants is a global concern, which is further intensified by the industrial and municipal wastewater discharge.

To address this challenge, the distillation and desalination processes have been extensively utilized for many years. Among various renewable resources, solar energy is regarded as one of the most effective and environmentally friendly options for desalination. The desalination technologies using solar energy includes multi-effect desalination^2^ multi-stage flashing^3^ solar driven reverse osmosis^4^ vapor compression desalination^5^ and electro-dialysis^6^.

Recent developments in solar-driven evaporation present valuable insights towards enhanced solar water desalination and recovery of resources. Zhang and Tan^7^ highlighted the necessity for standardized assessment methods in solar-water production studies for reporting consistent performance, and for fairly comparing the performance of different technologies. Further, Mao et al.^8^ discussed the development of solar steam generation, indicating a transition from efficiency-driven designs to multifunctional systems, that can enable sustainable water and energy solutions. Xiang et al.^9^ further reviewed solar-evaporation-based lithium extraction approaches, which offer insights into selective ion separation and about the role of evaporation structures in improving purification process. Recently, Liu et al.^10^^,^^11^ created a high-performance photothermal evaporator based on hierarchically ordered polypyrrole nanoarrays, which allow efficient movement of water in one direction, high evaporation rate, and selective crystallization. Their system highly concentrates lithium and rejects NaCl and has shown how the use of optimized solar absorbers and structured evaporators can be used to increase the efficiency of evaporation and control ions.

Along with solar-thermal desalination, atmospheric water harvesting (AWH) has also become viable solutions to the raising world-wide shortage of freshwater. Initially, hygroscopic substances were developed that could absorb and desorb moisture in the atmosphere efficiently. Nandakumar et al.^12,13^ have shown groundbreaking, hydrogel-based systems that could capture up to 420% of their weight in water, and releasing water passively by sunlight, with long-term cycling stability, collecting about 10 L kg^−1^day^−1^ without any external power input. By demonstrating sunlight-responsive water uptake and release for self-sustained irrigation devices, Yang et al.^14^ expanded AWH into agricultural automation. Mg-complex composite aerogels developed by Zhang et al.^15^ enabled full autonomy and scalability for atmospheric water production using sorption-based systems.

As a desalination strategy, interfacial solar evaporation (ISE) has made significant progress. The improvement in photothermal materials and evaporator designs has facilitated effective energy localization, salt rejection, and high solar-to-steam conversion efficiencies. For boosting freshwater yields, Wang et al.^16^ highlighted the importance of energy-management strategies, such as enthalpy-reduction and hierarchical evaporators. Subsequently, the scalable photothermal fabrics, vertically confined layers of water, and salt-insensitive evaporators with constant evaporation rates have been suggested in the pertinent literature^17,18^. Liu et al.^10,11^ demonstrates a groove-structured evaporator which could achieve 3.51 kg m⁻^2^ h⁻^1^ and simultaneously co-produce clean water, salt and electricity. In addition, material level study by Chen et al.^19^ show that solar thermal gradients and local heating play important roles in optimization of the evaporator performance.

Beyond desalination, solar energy satisfies various human requirements, such as drying vegetables^20–22^ heating air^23^ and generating electrical power^24^. The employment of photovoltaic (PV) panels necessitates a more intricate setup, increasing the cost of the process^25^. Therefore, there is a need for a desalination system that directly harnesses solar energy.

The solar still (SS) is a very popular and commonly used device to desalt the saline water because of its low-cost^26^ but its poor productivity limits its uses in the domestic and industrial sectors. Consequently, various approaches have been proposed to augment the efficiency and output of SSs. These methods include modifying their design, employing passive or active techniques, and incorporating heat storage materials such as sensible or latent heat storage^25,27–31^. To enhance productivity, various design alterations in SSs have been considered, such as single slope^32^ double slope^33^ tubular^34–36^ pyramid^37,38^ triangular^39^ spherical^40^ and tapered^41^. Additionally, improvements like cooling of the condensing cover^42,43^ adjustment of the condensing cover angle^44^ the use of reflectors^34,36^ fins^28,31,45^ a tracking mechanism^46^ wick^47^ and porous^48^ materials have also been explored.

Researchers have conducted experiments using various sensible heat storage materials (SHSMs) to augment the freshwater output of SSs. Arjunan et al.^49^ examined the impact of different SHSMs, such as pebbles, black granite gravel, and blue metal stone, on the effectiveness of conventional SS. The authors observed that black granite gravel surpassed pebbles and blue metal stones, achieving an efficiency of 43%. Samuel et al.^50^ conducted experimental research on SSs, using spherical balls and sponges as SHSMs. The productivity of SSs equipped with spherical balls and sponges increased by 68.18% and 22.7%, respectively, compared to conventional SS. Nasri et al.^51^ examined the effect of black gravels, sand, and black polythene on productivity using single slope SSs. Gravels and polythene demonstrated productivity improvements of 32.20% and 16.67%, respectively, compared to sand.

Kabeel et al.^52^ enhanced SS productivity by employing cement-coated red bricks as the SHSM. This modification increased the basin water temperature, the potential driving force for evaporation, by 34%, and consequently, productivity improved by 45% compared to CSS. To further enhance performance, Kabeel and Abdelgaied^53^ utilized a graphite absorber plate and implemented condensing cover cooling in a pyramid-shaped SS. These modifications led to remarkable improvements in productivity (107.7%) and efficiency (98.9%) compared to CSS. Economic analysis also revealed a 13.6% reduction in freshwater productivity cost. Further, Suraparaju et al.^54^ enhanced productivity by incorporating ball marble in the single-slope SS, resulting in a 22.8% improvement in productivity and a 36.7% boost in energy efficiency compared to CSS. Dhivagar et al.^55^ experimentally investigated single slope SS using crushed gravel sand as an SHSM, achieving improvements of 34.6% in productivity, 34.4% in energy, and 35% in exergy compared to CSS.

Thermo-economic analysis was performed by Sibagariang et al.^56^ on a double slope SS using oil palm shells. This study showed an improvement in energy efficiency by 41.71% and exergy efficiency by 2.994%, while productivity showed a 39% increase as compared to CSS. Panchal et al.^57^ conducted experiments on an active single slope SS with the addition of fins and pebbles. The authors observed significant improvements in productivity (144.1%), thermal efficiency (2.32%), and exergy efficiency (142%) compared to conventional SS. Moreover, the modified SS contributed to a reduction in CO_2_ emissions that was 2.44 times lower than conventional SS. In another effort to improve performance, Atteya and Abbas^58^ investigated stepped SSs using different sand beds. Their results revealed that the black sand stepped SS exhibited a remarkable 92% increase in productivity and a 49% improvement in daily efficiency over conventional SS.

Dumka et al.^59^ tested a modified SS that integrates Plexiglas and jute fabric for enhancing the heat localization and the thin-film evaporation. Their experimental findings revealed that the modified SS produced a distillate output 35% higher than the CSS. Moreover, the modified still resulted in a reduction of about 45% in the production cost of distillate output compared to the CSS. Pandey and Naresh^60^ investigated the feasibility of coupling pulsating heat pipes (PHP) with a pyramidal SS. The results revealed significant improvements in the yield (37.13% at a water depth of 3 cm), average energy efficiency (25.51% at a water depth of 2 cm) and average exergy efficiency (29.31% at a water depth of 2 cm) of the SS with PHP compared to CSS. Additionally, the cost per liter of fresh water decreased by up to 13.33% compared to the CSS (at water depth of 2 cm). Mahala and Sharma^61^ tested a novel pyramidal SS which uses fins and PCM inside the basin with and without gravels. Based on their experimental results, modified SSs with and without gravels produced higher daily productivity by 84% and 32%, respectively, than conventional SS. The modified solar still with gravels showed a significant improvement in energy efficiency (81.1%) and exergy efficiency (273 %) with a significantly shorter payback period (29%) and lower costs per liter of yield produced (29.2%) than conventional SS. Furthermore, Mahala and Sharma^62^ studied the impact of adding heat-storage and wick materials (gravels, chips, black cotton cloth, jute) on the yield of SSs. The modified solar still with gravels and cotton cloth configuration delivered the best results, surpassing CPSD with 64 % energy and 172 % exergy gains. Cotton-based distillers outperformed jute ones, achieving an 8 % higher productivity and stronger enviro-economic and carbon-mitigation metrics.

From existing literature, it becomes apparent that SSs, despite their affordability, exhibit low productivity. Consequently, researchers have investigated various design modifications and integrated SSs with heat storage materials, reflectors, fins, and heat pipes. These innovations have led to significant enhancements in SS efficiency and freshwater production compared to conventional stills (refer Table 1). The idea of using porous materials like clay pots for heat storage in SSs is inspired by traditional practices in rural areas, where pots are used to keep water cool during the summer. Clay pots have high porosity and thermal mass, enabling them to absorb and store heat effectively. In the context of SSs, this characteristic enhances thermal efficiency by providing steady heat release, which improves water evaporation and condensation rates. However, there has not been sufficient research conducted on utilizing clay pots as porous material inside a SBPSS to improve its efficiency and economic viability. Recently, Yarramsetty et al.^48^ reported that the use of clay pots as porous materials leads to a significant improvement (approximately 60%) in daily yield output. Taking a step forward, the present study explores the impact of various clay pot arrangements (facing upward, downward, and upward downward) on freshwater yield, energy efficiency, exergy efficiency, and economic analysis. Moreover, the impact of applying a black coating over the clay pots is also examined via thermo-exergo-economic analysis.

**Table 1:** Comparison of daily freshwater productivity achieved with various modifications in the pertinent literature.

References	Modification			Productivity, (L/m^2^)
Samuel et al.^50^	Single slope	+	• spherical ball salt storage • sponge	• 3.7 • 2.7
Dhivagar et al.^55^	Single slope	+	• crushed gravel sand heat storage and biomass evaporator	• 34.6% improvement
Essa et al.^34^	Tubular	+	• Jute cloth with nanocomposites	• 9.0
Elmaadawy et al.^33^	Double slope	+	• Gravels, black wick, dispersed carbon black nanoparticles	• 68% improvement
Sharma et al.^28^^,^^31^	PSS	+	• Cemented blocks • Black-coated cemented blocks	• 1.2 • 1.36
Sharma et al.^28^^,^^31^	Triangular	+	• sand • bricks • bricks with napkins	• 3.12 • 2.08 • 2.29
Kumar et al.^43^	Single slope	+	• Uncoated cylindrical cement fins • Black-coated cylindrical cement fins • Black-coated cylindrical cement fins wrapped in black cotton cloth	• 3.3 • 3.8 • 4.44

The paper is structured as follows: Section 2 describes the experimental setup and methodology. Section 3 evaluates the performance metrics, including energy, exergy, economic and environmental analyses. Section 4 presents the findings, covering the experimental observations, freshwater productivity, energy efficiency, exergy efficiency, economic assessment, environmental impact and performance indexes comparison. Finally, Section 5 summarizes the key findings and suggests directions for future research.

## Experimental facility and procedure

**Fig. 1:**
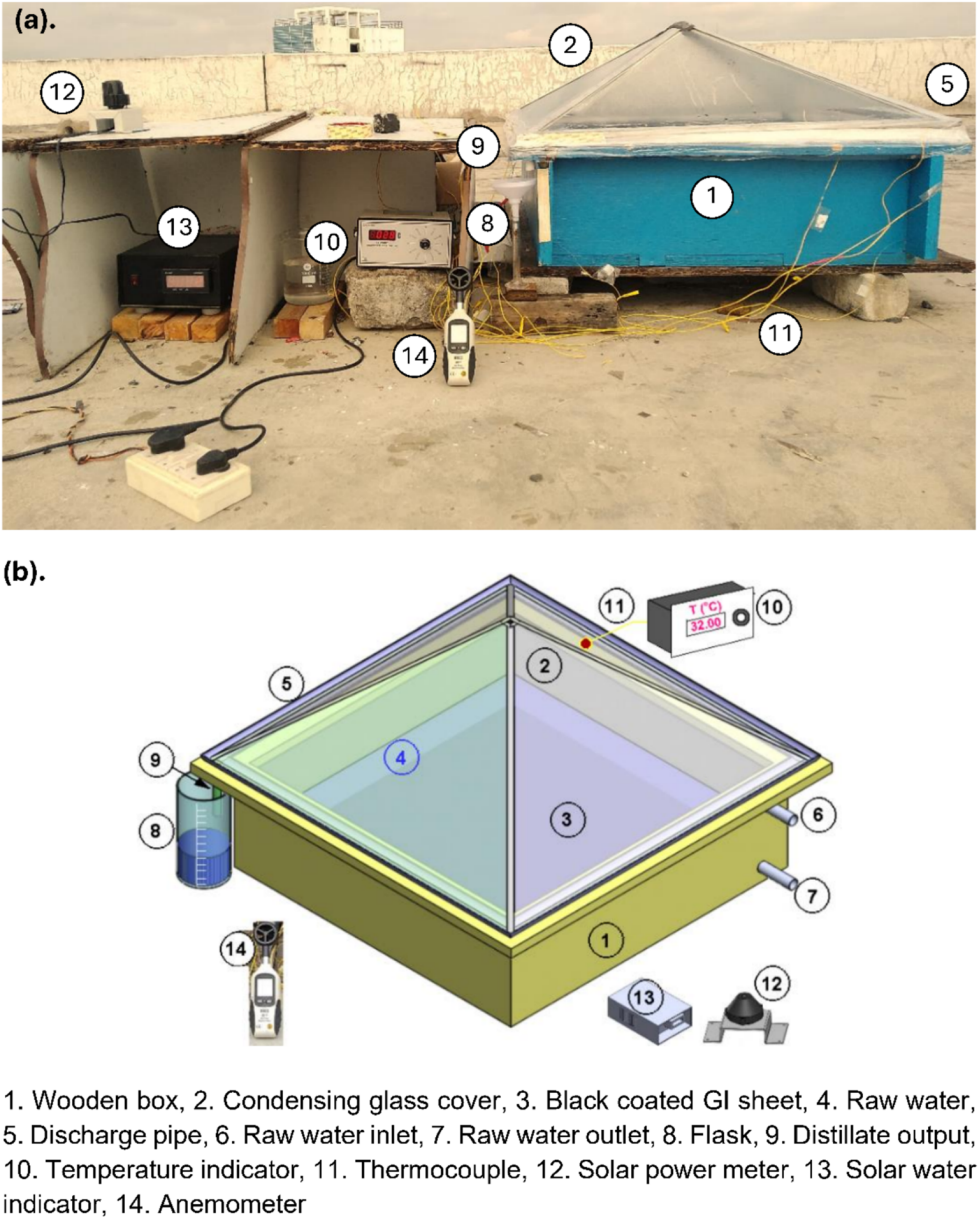
Developed experimental facility with instruments for experimentation: (**a**). Photograph, and (**b**). 3-D model.

The developed experimental system, consisting of a SS in pyramid form as depicted in Fig. 1 (a) and (b), was positioned on the roof of a standalone building having coordinates 16.6834°N, and 80.3904°E. The SS is built with robust wood (thickness of 0.022 m) that provides both a strong framework and insulation to reduce thermal loss to the environment. Its inner structure is covered with a 2-mm thick galvanized iron (GI) sheet, which acts as a barrier between the wood and the brine. To enhance the heat absorption and speed up the evaporation process, a layer of black paint is applied to the GI sheet.

The solar distiller basin possesses dimensions of 710 mm in length, 710 mm in breadth, and 220 mm in height, providing an absorbing area of 0.5041 m^2^. Additionally, a condensing cover, designed in form of a pyramid, made of 3-mm glass having transmissivity of 0.9, is placed at an angle approximately equal to 17°. This pyramid shape of the condensation glass cover offers increased surface area for vapor condensation. The solar still is equipped with a railing mechanism that allows smooth movement of the pyramidal glass cover, enabling easy and regular access to the SS basin. This structural modification allows the regular cleaning of the glass cover and basin before the experiments begin and thereby effectively prevents the accumulation of salt crystals and dusts on both the basin liner and the inner surface of the glass cover.

A trough positioned over the basin, connected to a distillate collecting beaker via a flexible hose, collects the distillate produced during the "evaporation-condensation process." It is important to note that to prevent any potential leakage, the junction between the basin and the condensing cover was sealed using silicon and m-seal as bonding materials.

**Fig. 2:**
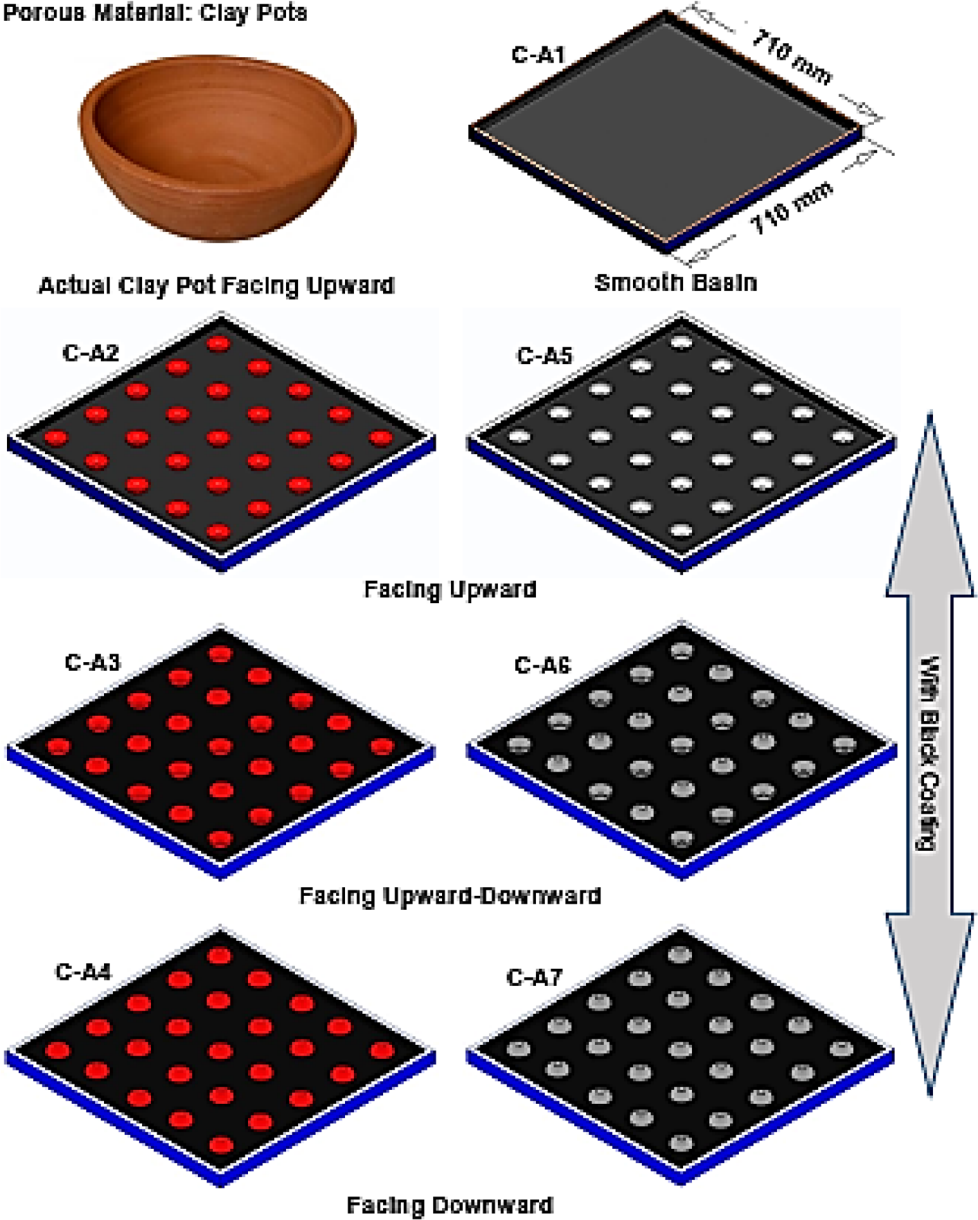
Configurations under consideration to enhance productivity.

To enhance the evaporation of raw water, the PSS absorber basin is equipped with clay pots with and without black coating (refer to Fig. 2). The clay pots (average diameter of 50 mm and height of 20 mm), that were discarded after use during Diwali festival, were selected as a SHSM in the experiments. Typically, the handmade clay pots (i.e. diya) have an open porosity of about 20–30%, pore-size distribution of about 10–40 µm, an average wall thickness of about 4 mm and an average water absorption capacity of 15–20% by weight. Strategic placement of these clay pots is anticipated to significantly increase freshwater yield. The assessment of the SS’s performance was conducted for these cases, while upholding a consistent water level of 30 mm.

K-type thermocouples (with a temperature range of 0−1250 °C with an accuracy of ± 1 °C) along with a digital temperature indicator was employed to monitor and record temperatures at various points, including the absorber wall, salty water, vapour, inner glass cover, and ambient temperatures. The experimental setup also included measurements of global radiation and wind speed for the days of experimentation, utilizing a Pyranometer (0−1800 W/m^2^ ± 5 W/m^2^) and an anemometer (0–25 m/s), respectively.

To initiate the process, 30 mm of tap water is added to the SS. As the sun rises, solar radiation goes into the SS through pyramidal glass cover, and the water absorbs heat. Adequate energy absorption leads to the release of water vapor during the heating phase. The generated water vapour condenses over the condensing cover due to temperature fluctuations at the glass surface. The condensed water droplets then travel along the inclined glass and is gathered in a jar. In SSs, water purification takes place through the combined processes of evaporation and condensation, thereby producing clean water that can be used for household applications.

The present work has some limitations. This research was conducted in Andhra Pradesh (16.6834°N, and 80.3904°E), so the weather conditions may not be the same as in other regions. The tests were only done on clear sky days, which could affect how the results apply to different weather conditions. Lastly, the experiments were carried out between 7:00 AM and 7:00 PM, so they might not account for changes in solar radiation outside these hours.

Considering potential instrumental errors, the errors associated with hourly and overall freshwater yield are approximately 1.5% and 2.5%, respectively. Moreover, errors in solar radiation intensity and temperature measurements amount to 2.88% and 0.58%, respectively. As a result, the cumulative uncertainty in determining the energy and exergy efficiencies of the SBPSS reaches 3.0% and 2.85%, respectively.

## Performance assessment

### Energy efficiency

The SS’s energy efficiency, which is the ratio of thermal energy transformed into distilled water to the solar energy received over a specific period, serves as an indicator of its thermal performance. Energy efficiency of the SS (${\eta }_{en}$) is calculated using Equation (1), where ${\dot{m}}_{w}$

represents the daily freshwater output (kg/day), ${A}_{b}$​ denotes the SS’s absorber area, ${I}_{s}$​ indicates the solar irradiation, $\Delta t$ refers to the time duration, and ${h}_{fg}$ is the latent heat of vaporization (J/kg.K), which can be determined by Equation 2^28,31^.

1 $${\eta }_{en}=\frac{{\dot{m}}_{w}\times {h}_{fg}}{{A}_{b} {I}_{S} \Delta t}\times 100$$

2 $${h}_{fg}=2.4935\times 1{0}^{6}\times \left[1-9.4779\times 1{0}^{-4} {T}_{w}+1.3132\times 1{0}^{-7} {T}_{w}^{2}-4.794\times 1{0}^{-9} {T}_{w}^{3}\right]$$

### Exergy analysis

To assess the amount of energy available for a specific application, like a SS, the exergy efficiency (${\eta }_{ex}$) is calculated. Exergy reflects the potential work achievable by the SS once it reaches a state of thermodynamic equilibrium. It can be calculated as follows^61^.

3 $${\eta }_{ex}=\frac{{E}_{ex,out}}{{E}_{ex,in}}$$

Where, ${E}_{ex,in}$ and ${E}_{ex,out}$ denote the input and output exergy, which are determined using Equation (4) and Equation (5) respectively^61^.

4 $$\sum {E}_{ex,in}={I}_{S}\times {A}_{b}\times \left[1-\frac{4}{3}\left(\frac{{T}_{a}}{{T}_{sun}}\right)+\frac{1}{3}{\left(\frac{{T}_{a}}{{T}_{sun}}\right)}^{4}\right]$$

5 $$\sum {E}_{ex,out}={\dot{m}}_{w}\times {h}_{fg}\times \left[1-\left(\frac{{T}_{a}}{{T}_{w}}\right)\right]\times \frac{1}{3600}$$

Where, ${T}_{a}$ and ${T}_{sun}$ represent the temperatures of the ambient and the sun, respectively.

### Economic analysis

Assessing the economic viability of a solar desalination system is crucial for determining its profitability and practicality for the consumer. Table 2 presents a comprehensive analysis of the fixed costs associated with SS under various conditions, providing a deeper insight into their financial components. The overall cost of producing freshwater from saline/raw water via a SS is influenced by the expected service life of SS, the annual days of operation, and the current interest rate, as presented in Table 3, in addition to annual yield (${M}_{a}$), initial fixed cost, and ongoing maintenance cost. Furthermore, the equations used for the economic assessment are summarized in Table 4.

**Table 2:** Fixed cost (in Rs.) for the evaluated configurations.

Component of PSS	Smooth basin	Configurations with clay pots	Configurations with black coated clay pots
	C-A1	C-A2, C-A3, and C-A4	C-A5, C-A6, and C-A7
Wooden box	1500	1500	1500
Absorber (GI) sheet	2000	2000	2000
Metal frame	1500	1500	1500
Condensing cover (Glass)	2000	2000	2000
Collecting Jar	370	370	370
PVC pipe	100	100	100
Black paint	470	470	770
Clay pots	-	160	160
**Total fixed cost (**${F}_{c}$**)**	**7940**	**8100**	**8400**

**Table 3:** Key assumptions and their values used for economic analysis.

Variable	Mean, Unit	Value
$i$	Interest rate, %	10
$n$	Expected service life, Years	15
$N$	Days of operation, Day/Year	330
${M}_{c}$	Market cost of freshwater per Liter, Rs.	20

**Table 4:** Equations applied in the economic analysis^28^^,^^31^^,^^61^.

Description	Equation	No.
Capital recovery factor	$CRF=\frac{i\times (1+i{)}^{n}}{{\left(1+i\right)}^{n}-1}$	(6)
Annual fixed cost	$FAC=CRF \times {F}_{c}$	(7)
Annual maintenance cost	$AMC=0.1\times FAC$	(8)
Sinking fund factor	$SFF=\frac{i}{{\left(i+1\right)}^{n}-1}$	(9)
Annual salvage value	$ASV=0.2 \times {F}_{c}\times SFF$	(10)
Total annual cost	$TAC=FAC+AMC-ASV$	(11)
Cost of fresh water	$CPL=\frac{TAC}{{M}_{a}}$	(12)
Payback period	$PP=\frac{\text{Initial fixed cost}}{\text{Annual earning}}\times 365$	(13)

### Environmental analysis

The carbon dioxide (CO_2_) emissions for the investigated SBPSS cases over their lifespan can be evaluated as follows^61^:

14 $${CO}_{2}\text{ emission}= \frac{2.0 \times \text{Embodied energy}}{\text{Lifespan of SBPSS} }$$

The net CO_2_ emission mitigation, an environmental parameter, over the lifespan of SBPSS and is evaluated as follows^61^:

15 $${CO}_{2} \text{emission mitigation}= \frac{\left(\left(\text{Energy output}\times \text{Lifespan of SS}\right)- \text{Embodied energy}\right)\times 2.0}{1000}$$

The carbon credits accumulated through the reduction of CO_2_ emissions over the entire lifespan of investigated SBPSS cases is evaluated as^61^:

16 $$\text{Carbon credits accumulated}= {CO}_{2} \text{emission mitigation}\times \text{Trading cost of carbon}$$

## Results and discussion

In this study, experimental data of temperatures at strategically chosen locations and freshwater yield were measured from 7:00 AM to 7:00 PM on hourly basis to assess the performance of SS. Further, the performance indexes, i.e. productivity, energy efficiency, exergy efficiency, and economic aspects, of SSs using porous material with and without black coating (designated as C-A2 to C-A7) were compared with those of a CSS (C-A1) under identical environmental conditions.

### Experimental observation

**Fig. 3:**
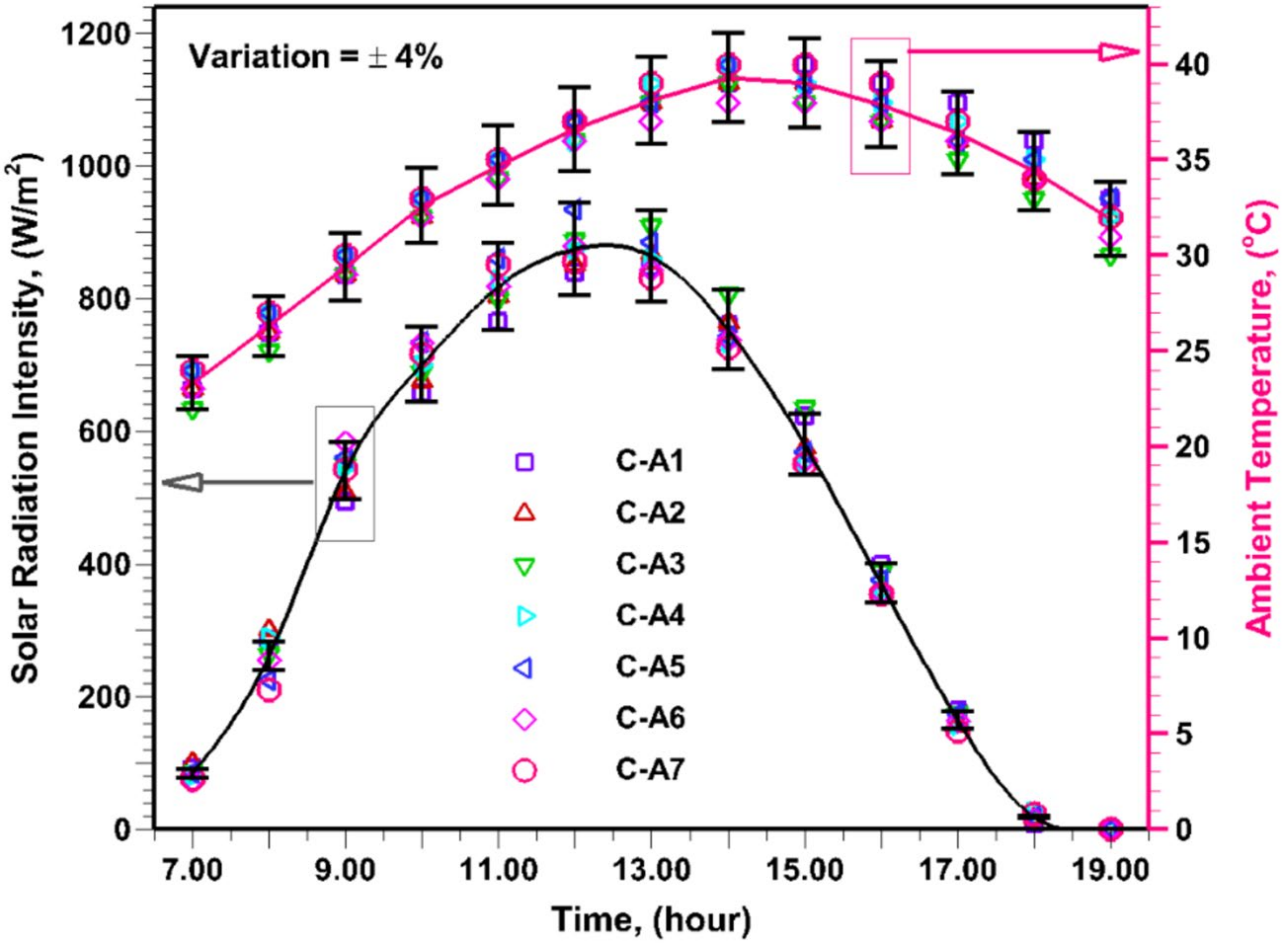
(**a**) Solar intensity and (**b**) ambient temperature variation for experimental days.

Fig. 3 illustrates the hourly variation of solar irradiance for the investigated cases throughout the experimental duration. As anticipated, solar radiation intensity gradually increases from 7:00 AM, getting its peak value (~880 W/m^2^) at 12:00 PM, and subsequently decreases, attaining its lowest value at 6:00 PM due to sunset. The trend of ambient temperature variation is like solar radiation intensity; however, a time lag is observed between the peak temperature and solar irradiance values, owing to the time needed to warm the different components of SBPSS. In the afternoon, the maximum average ambient temperature reached approximately 39.3°C, while in the evening, it decreased to around 31.9°C. The variation in solar irradiance and ambient temperature for the experimental days falls within a 4% range, allowing a meaningful comparison of the experimental outcomes for evaluating the ability of porous material with and without black coating in augmenting the performance of SBPSS (Fig. 3).

**Fig. 4:**
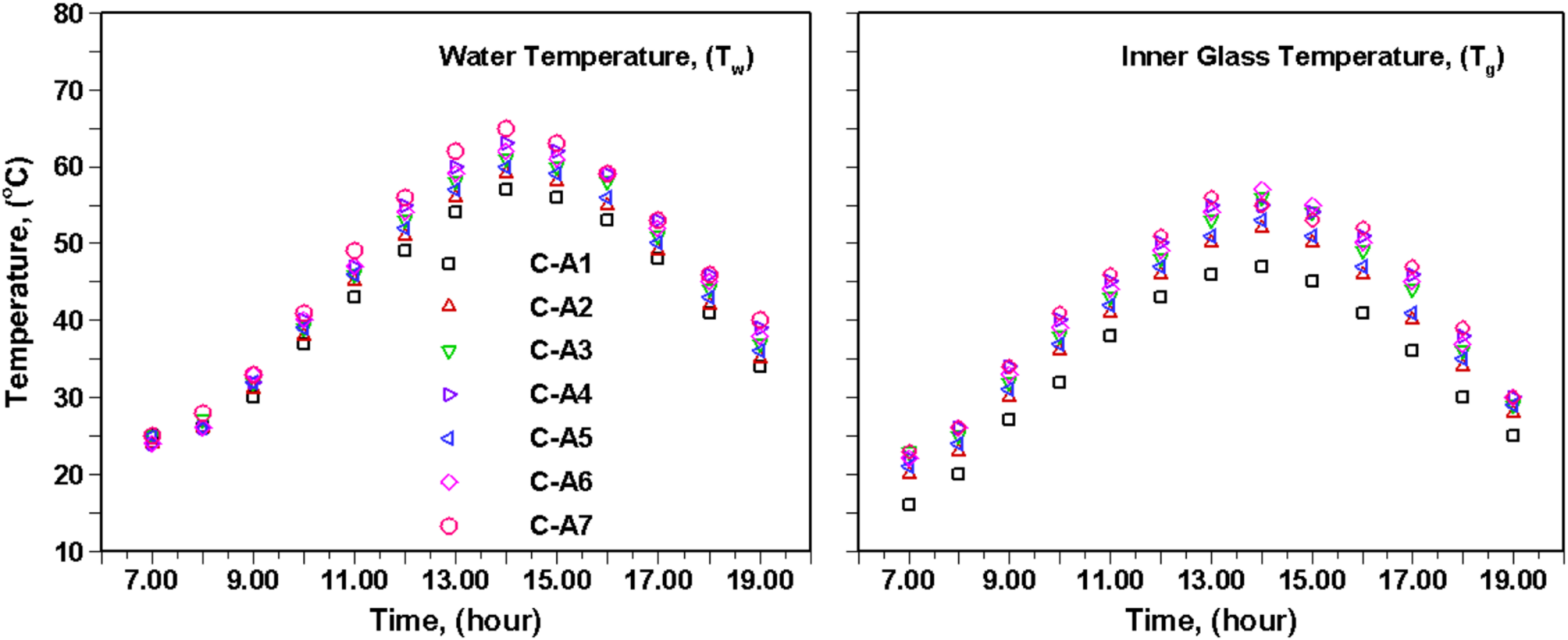
Comparison of temperature inside SBPSS for investigated cases.

Fig. 4 presents the temperatures of raw water and the condensing glass cover inside the SS during the experimentation. These temperatures are crucial for understanding the yield calculation for different configurations shown in Fig. 2. Initially, both the temperatures rise, reaching their zenith, followed by a subsequent decline. Notably, the maximum glass temperature for configurations C-A1, C-A2, C-A3, C-A4, C-A5, C-A6, and C-A7 was approximately 47 °C, 52 °C, 56 °C, 55 °C, 53 °C, 57 °C, and 56 °C, respectively. While, the maximum water temperatures for the same configurations were reported as 57 °C, 59 °C, 61 °C, 63 °C, 61 °C, 63 °C, and 66 °C, respectively. The water temperature exhibited significant improvement, approximately 11% for C-A4, due to the impact of clay pots compared to water temperature of CSS. This is because using porous materials, such as clay pots, increases the surface area, allowing for better heat absorption and retention. Consequently, this leads to a higher water temperature inside the still, resulting in more efficient evaporation and a higher yield of freshwater. Additionally, this improvement was further increased to 16% (for C-A7) with the black coating over the clay pots than the water temperature of C-A1. Evidently, the inclusion of porous material, both with and without black coating, in the SS significantly raised the water temperature compared to C-A1. The higher difference between water and glass temperatures resulted in improved condensation, leading to higher yield in configurations C-A2 to C-A7 when compared to C-A1.

### Fresh water productivity

**Fig. 5:**
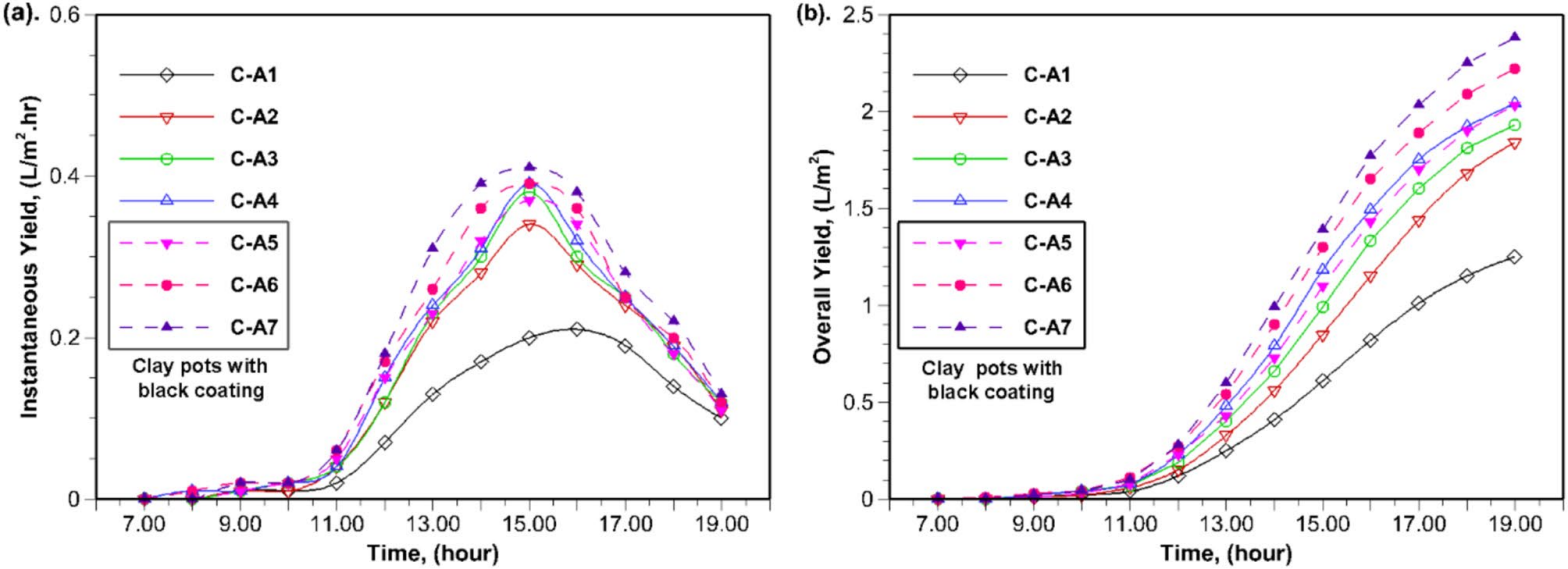
Hourly variation of (**a**) instantaneous yield and (**b**) cumulative yield for investigated configurations.

The hourly freshwater yield and cumulative yield for the investigated configurations (C-A1 to C-A7) are depicted in Fig. 5(a) and (b) respectively. The instantaneous yield initially increases, reaching maximum values, and is subsequently followed by a decline. This trend holds true regardless of the specific configurations being investigated (Fig. 5(a)). The observed variations in yield are associated with changes in solar radiation intensity and ambient temperature during the experimentation. In the morning hours, there was no significant effect of porous material on the yield. However, after 11:00 AM, the hourly productivity for configurations with and without black-coated porous materials improved significantly compared to C-A1. Among the configurations with clay pots, the maximum instantaneous yield of approximately 0.39 L/m^2^ was observed for C-A4 at 3:00 PM due to the heat energy retained by the clay pots. Meanwhile, in clay pots with black coating, the maximum instantaneous yield of about 0.41 L/m^2^ was observed for C-A7. Among the tested configurations, C-A7 produces the highest cumulative yield, followed by C-A6 for the entire duration of the experiments than others, as seen in Fig 5.

**Fig. 6:**
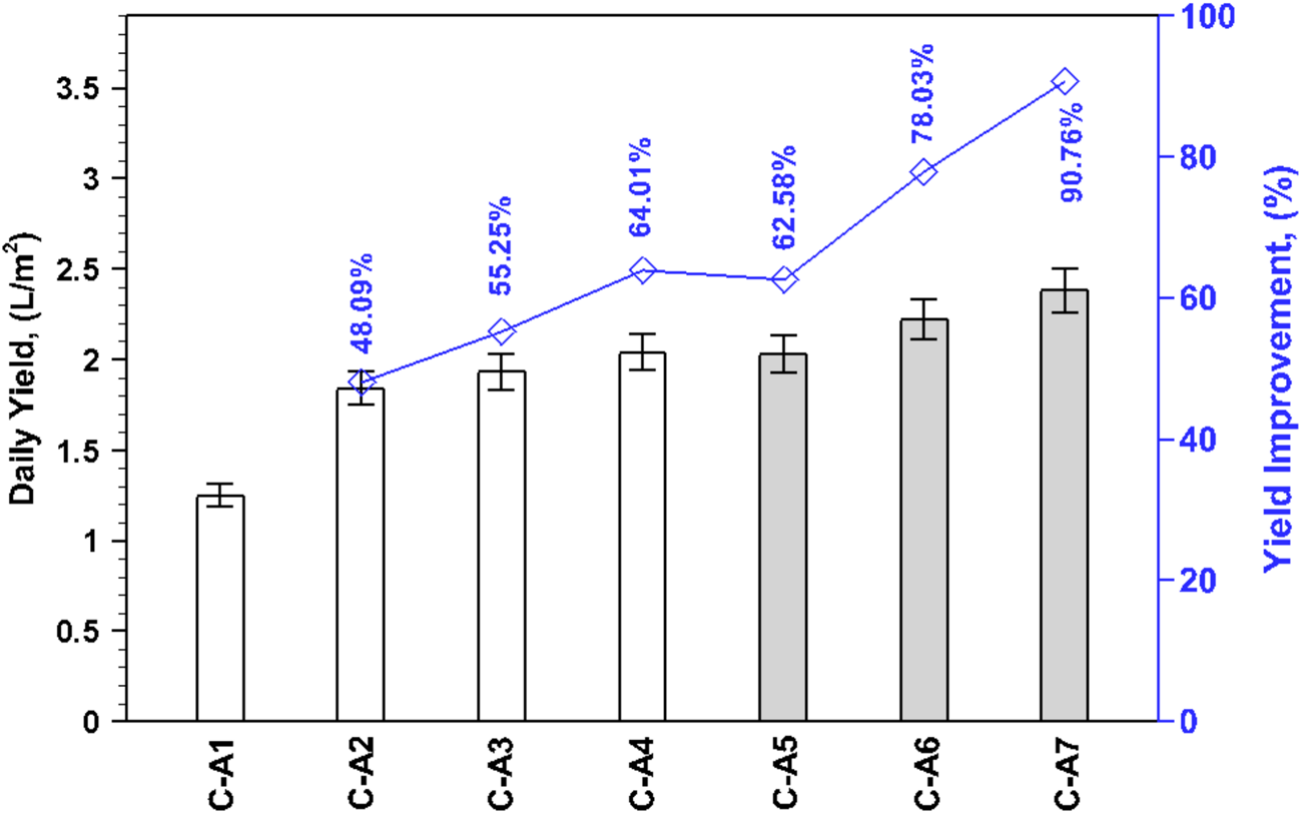
Comparison of cumulative yield and yield improvement for investigated cases.

The cumulative yield for the investigated cases is compared in Fig. 6. As expected, the inclusion of clay pots, both with and without black coating, has a pronounced effect on the overall yield. The daily yield of 1.84, 1.93, 2.04, 2.03, 2.22, and 2.38 L/m^2^ has been observed for configurations C-A2, C-A3, C-A4, C-A5, C-A6, and C-A7, respectively, in comparison to the cumulative yield of 1.25 L/m^2^ for C-A1. Notably, the freshwater yield is 48.09%, 55.25%, and 64.01% higher when the clay pots face upward (C-A2), upward-downward (C-A3), and downward (C-A4), respectively, compared to the yield without clay pots (C-A1). Evidently, the freshwater yield is further augmented by applying black coating over the clay pots: 62.58% for clay pots facing upward (C-A5), 78.03% for clay pots facing upward-downward (C-A6), and an impressive 90.76% for clay pots facing downward (C-A7).

It is important to highlight that when the clay pots are facing downward (C-A4), the effect of porosity dominates, resulting in an 11% higher yield than pots facing upward (C-A2). Interestingly, the yield improvement is further enhanced to about 29.35% by applying black coating to the downward-facing clay pots (C-A7) compared to C-A2. Compared to porous material alone, applying a black coating over the porous material results in approximately a 10–17% increase in freshwater yield, depending on the arrangement of the clay pots.

### Energy efficiency

**Fig. 7:**
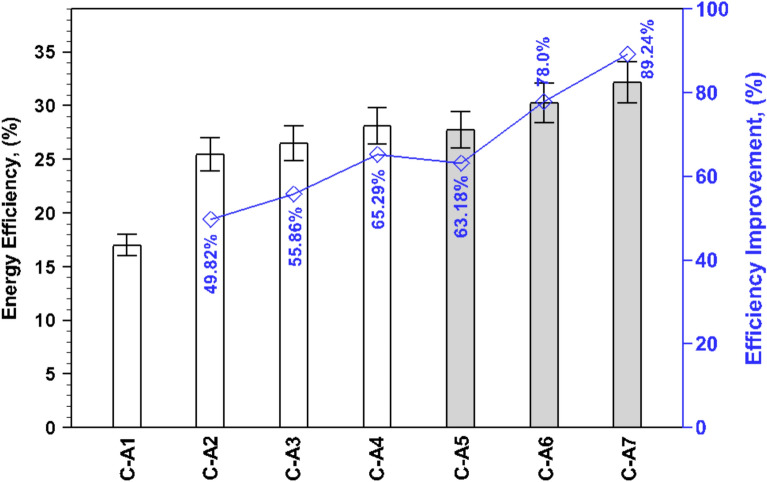
Comparison of energy efficiency for investigated cases.

Fig. 7 presents a comparison of energy efficiency, calculated using Eq. (1), for all the studied cases. From Eq. (1), it is evident that energy efficiency strongly lie on the power of solar intensity and the freshwater yield for a constant basin area. The overall energy efficiency of C-A1, C-A2, C-A3, C-A4, C-A5, C-A6, and C-A7 is 17%, 25.47%, 26.49%, 28.10%, 27.74%, 30.29%, and 32.17%, respectively.

The use of clay pots in the SBPSS leads to an improvement in energy efficiency ranging between 49.82% and 65.30%, depending on the arrangement of the clay pots. This improvement is further intensified with the use of black coating over the clay pots, resulting in efficiency values between 63.22% and 89.26%. This indicates the significant impact of black coating on porous material in enhancing the energy efficiency of the SBPSS. The improved energy efficiency is attributed to the effect of clay pots, both with and without black coating, on water temperature (see Fig. 4). The higher water temperature leads to increased freshwater yield (as seen in Fig. 6), which in turn contributes to better energy efficiency. Among the studied cases, the highest energy efficiency is achieved when clay pots face downward (C-A4), reaching 28.10% for porous material only. When combined with black coating, this efficiency further increases to 32.17% for C-A7.

### Exergy efficiency

**Fig. 8:**
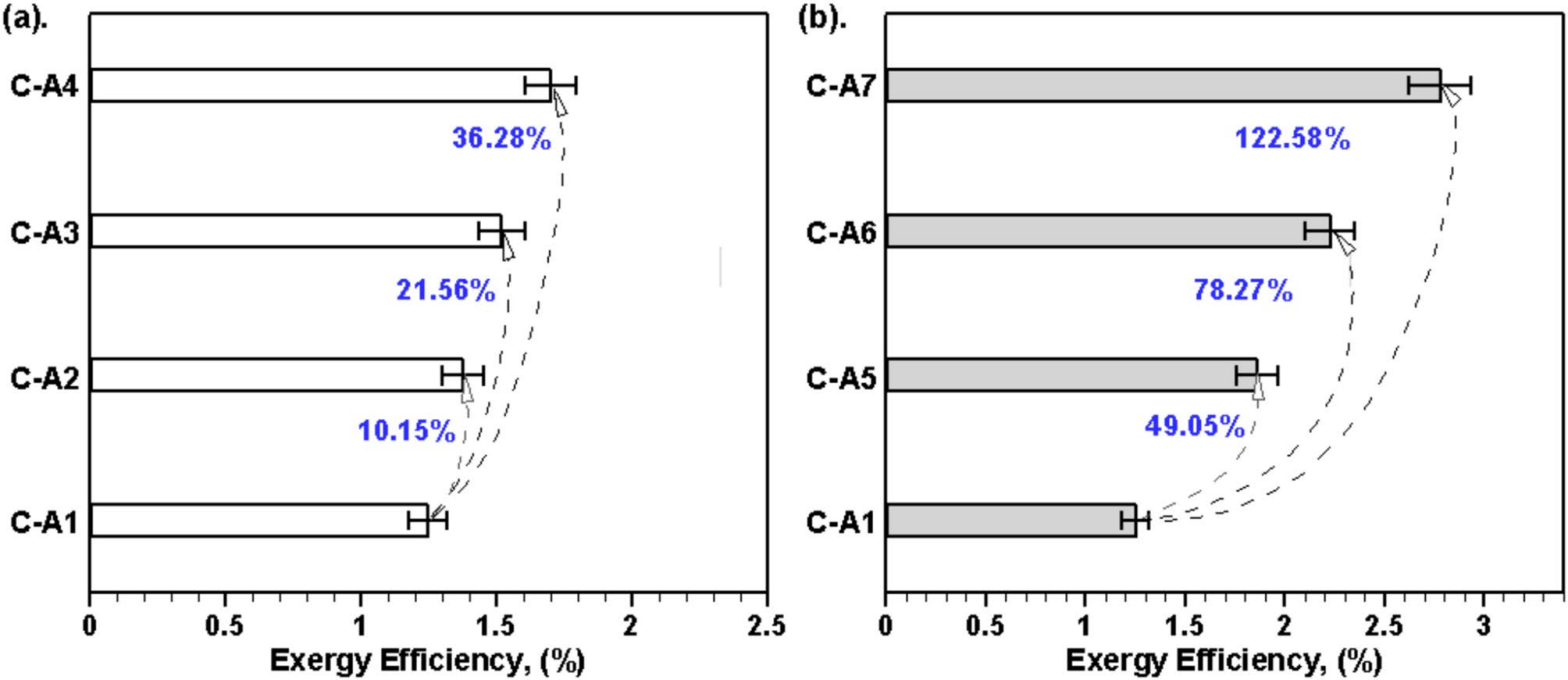
Comparison of exergy efficiency for C-A1 to C-A7 cases.

**Fig. 9:**
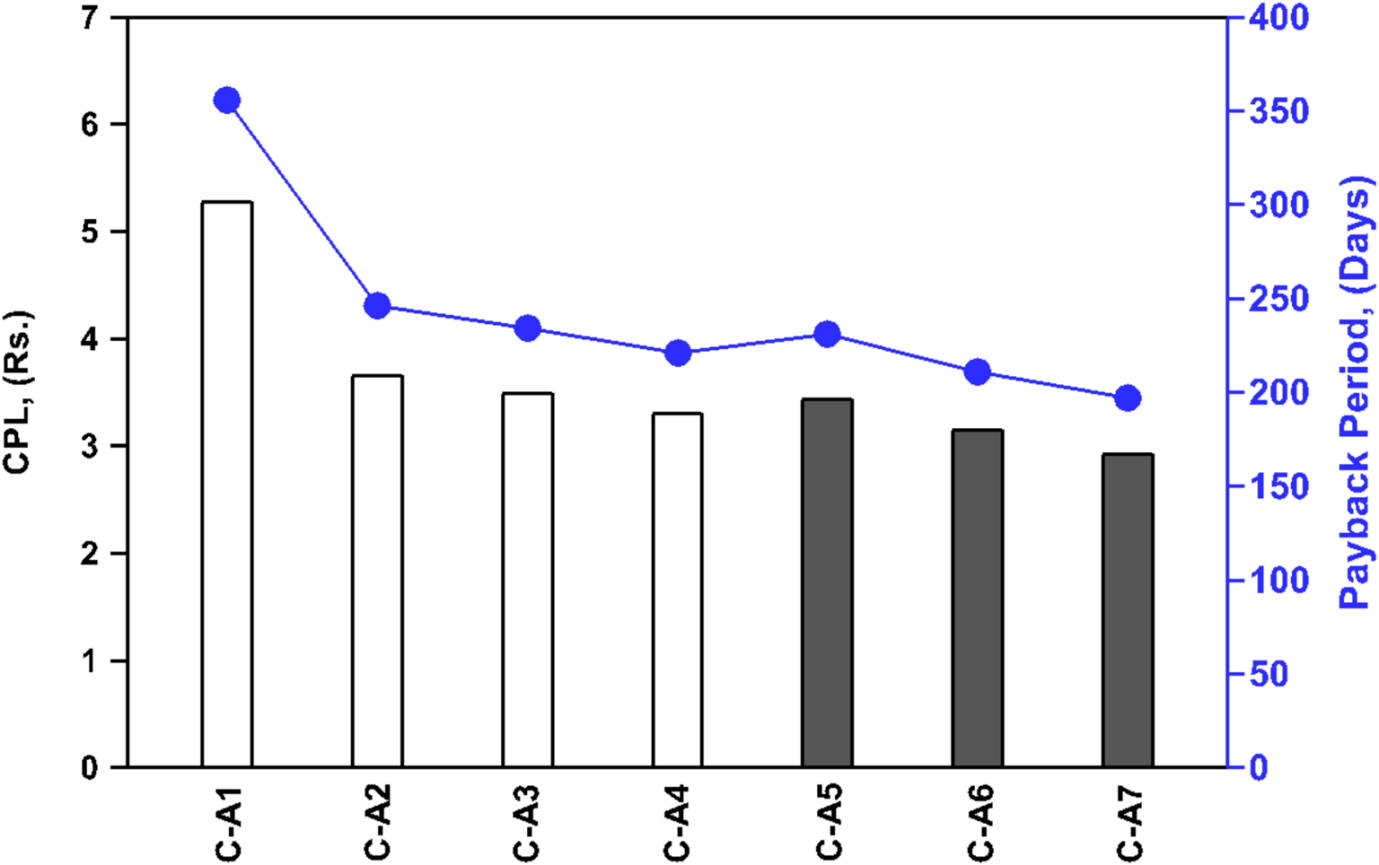
Comparison of CPL and payback period for investigated cases.

Exergy efficiency plays a key role in determining how well a system can convert available energy into work. Fig. 8 illustrates the profound impact of using porous materials and black coatings on the exergy efficiency of a SBPSS. The use of clay pots in SSs (C-A2, C-A3, and C-A4) results in increased exergy efficiencies of 1.38%, 1.52%, and 1.70% respectively, compared to the smooth SBPSS (C-A1), which has an exergy efficiency of 1.25% (see Fig. 8(a)). The rising temperature of the water due to the inclusion of clay pots is mainly responsible for the increased exergy efficiency and consequently leading to improved performance for C-A2 to C-A4 configurations. The increase in exergy efficiency for SSs with clay pots (C-A2 to C-A4) is approximately in the range of 10.15% to 36.28% compared to the baseline (C-A1), thus, demonstrating the ability of clay pots in improving the exergy efficiency of SSs. The analysis of Fig. 9(b) shows that coating the clay pots with a black material, which increases their heat absorption capability, leads to even higher exergy efficiencies. For C-A5, C-A6, and C-A7, the exergy efficiencies are 1.86%, 2.23%, and 2.78% respectively. The substantial improvements in exergy efficiency for these configurations over C-A1, 49.05% for C-A5, 78.27% for C-A6, and 122.58% for C-A7, highlight the significant impact of black coatings on the thermal dynamics of the system.

### Economic assessment

Table 5 presents the estimated values of various cost parameters in economic assessment of considered SS configurations. Table 5 reveals that incorporating clay pots on the absorber plate significantly boost the average yearly yield (${M}_{a}$), up to 260.7 L/m^2^ for C-A4, Additionally, applying a black coating to the clay pots further increases the yearly yield by approximately 17% for configuration C-A7 compared to C-A4.

**Table 5:** Cost parameters estimated for configurations under investigation.

Configurations	$FAC$ (Rs.)	$AMC$ (Rs.)	$ASV$ (Rs.)	$TAC$ (Rs.)	${M}_{a}$ (L/m^2^ year)	$CPL$ (Rs./L)
C-A1	1044	104	50	1098	412.5	5.28
C-A2	1065	106	51	1120	607.2	3.66
C-A3	1065	106	51	1120	636.9	3.49
C-A4	1065	106	51	1120	673.2	3.30
C-A5	1104	110	53	1162	669.9	3.44
C-A6	1104	110	53	1162	732.6	3.15
C-A7	1104	110	53	1162	785.4	2.93

Although initially, there were concerns about the extra costs due to the addition of porous materials and black coatings, the overall cost per liter (CPL) has seen a substantial decrease as evidenced from Fig. 9 and Table 5. The addition of porous materials has led to a CPL of Rs. 3.66 for C-A2, Rs. 3.49 for C-A3, and Rs. 3.30 for C-A4, which is a remarkable drop of up to 37.5% for C-A4 when compared with C-A1. Moreover, the application of a black coating on the porous materials results in a slight increase in cost but also leads to a significant CPL reduction of about 44.5% for C-A7 compared to C-A1. This notable decrease in CPL reflects a major boost in productivity against the investment, thus confirming the cost-effectiveness of our strategy. Based on an average cost of fresh water Rs. 20 for a liter in our local market, the net annual profit generated by C-A4 is around Rs. 13358, which is increased to Rs. 15598 for C-A7 where black coating applied to the clay pots.

Additionally, Fig. 9 reveals significant variations in payback periods for different configurations of SSs, i.e. from C-A1 to C-A7. The smooth SBPSS (C-A1) has a payback period of approximately 356 days, serving as a baseline for comparison. In contrast, the payback periods for SSs incorporating clay pots are 246 days (C-A2), 234 days (C-A3), and 221 days (C-A4), indicating that the addition of clay pots substantially enhances the cost-effectiveness of the system. The introduction of black-coated clay pots, which absorb and retain heat more effectively, leads to even shorter payback periods of 231 days (C-A5), 211 days (C-A6), and 197 days (C-A7). There is a maximum reduction of about 37.9% in the payback period for SBPSS with clay pots (C-A4), while this further improves to about 44.7% for SBPSS with black coating on clay pots (C-A7), making the investment even more attractive.

In summary, SBPSS integrated into clay pots have higher annual yields and lower CPL, and black coating further enhances these benefits. This modification reduces the overall CPL substantially, confirming its cost-effectiveness despite initial concerns. By adding clay pots and black coatings to SSs, payback periods are also reduced considerably, making these configurations more economically viable. Overall, the modifications lead to higher profitability and productivity.

### Environmental impact

The SBPSS operate without emitting CO_2_. However, manufacturing their components, like the aluminum channel, GI absorber, and glass cover, requires electrical energy, mainly from fossil fuels. This energy use, known as embodied energy, helps evaluate environmental impacts such as CO_2_ emissions, CO_2_ mitigation, and carbon credits accumulated, as tabulated in Table 6. The embodied energy, as estimated using the method from our earlier publication^61^, is 110 kWh for C-A1, 114 kWh for C-A2, C-A3, and C-A4, and 118 kWh for C-A5, C-A6, and C-A7.

**Table 6:** Environmental parameters of solar distiller for life cycle of 15 years.

Environmental parameters	C-A1	C-A2	C-A3	C-A4	C-A5	C-A6	C-A7
CO2 emission (kg/year)	11.59	12.01	12.01	12.01	12.43	12.43	12.43
CO_2_ mitigation (tons/year)	3.65	6.15	5.56	5.79	7.06	6.06	6.63
Carbon credits accumulated (Rs.)	3069.90	5164.46	4666.90	4861.11	5929.79	5092.28	5568.50

The CO_2_ emissions for the seven cases range from 11.59 kg/year to 12.43 kg/year (refer Table 6). The lowest emission is observed in C-A1 (11.59 kg/year), while the highest emissions are seen in C-A5, C-A6, and C-A7 (12.43 kg/year). This indicates that C-A1 is the most environmentally friendly in terms of CO2 emissions, while C-A5, C-A6, and C-A7 have the highest emissions. Furthermore, the CO_2_ mitigation measures the amount of CO_2_ reduced per year. The data shows that C-A5 has the highest CO_2_ mitigation at 7.06 tons/year, followed by C-A7 at 6.63 tons/year. The lowest mitigation is observed in C-A1 at 3.65 tons/year. This suggests that C-A5 is the most effective in reducing CO_2_ emissions, up to 93.42% higher than C-A1. The carbon credits accumulated represent the economic value of the CO_2_ mitigation efforts. The values range from Rs. 3069.90 to Rs. 5929.79 (refer Table 6). C-A1 has the lowest value (Rs. 3069.90), while C-A5 has the highest value (Rs. 5929.79). This indicates that C-A1 is the least economically beneficial in terms of carbon credits, whereas C-A5 is the most beneficial. Overall, this data highlights the trade-offs between environmental impact and economic benefits. While some cases like C-A1 are environmentally friendly with low emissions, they may not be as effective in mitigating CO_2_ or generating carbon credits. On the other hand, cases like C-A5, despite having higher emissions, offer higher CO_2_ mitigation and economic benefits through carbon credits. Decision-makers need to balance these factors based on their priorities and goals.

### Performance indexes comparison

Table 7 illustrates the enhanced productivity of our current study compared to relevant literature on SSs using various heat storage materials. The comparison highlights the significance of employing porous materials, both with and without black coating, in SBPSSs. Undoubtedly, employing clay pots—both with and without black coating—as porous materials lead to favourable results in relation to freshwater production, efficiency, and economic viability, with shorter payback periods.

**Table 7:** Comparison of percentage improvement in yield, energy and exergy efficiency, and percentage reduction in *CPL* and payback period of present study with pertinent literature.

References	Modification			Improvement (%)			Reduction (%)	
				${\dot{m}}_{w}$	${\eta }_{en}$	${\eta }_{ex}$	$CPL$	$PP$
Samuel et al.^50^	Single slope	+	• spherical ball salt storage • sponge	54.2 08.3	-	-	33.3 06.7	-
Panchal et al.^45^	Single slope	+	• inclined fins • vertical fins	26.8 24.2	-	-	-	-
Kabeel et al.^52^	Single slope	+	• coated red bricks	60.0	-	-	45.1	43.9
Suraparaju et al.^54^	Single slope	+	• Ball marbles	21.2	-	-	8.0	12.3
Elmaadawy et al.^33^	Double slope	+	• Gravels, black wick, dispersed carbon black nanoparticles	68	50.6	146.3	30.7	-
Yarramsetty et al.^48^	PSS	+	• upward facing clay pots • downward facing clay pots	47.2 60.0	-	-	-	-
Panchal et al.^57^	Single slope	+	• evacuated tubes solar collector, perforated fins, and pebbles	144	2.3	142.2	8.9	-
Atteya and Abbas^58^	Single slope	+	• stepped • yellow sand • black sand with reflector	55 71 92	24.4 31.4 41.2	-	-	-
Pandey and Naresh^60^	PSS	+	• pulsating heat pipe	37.1	25.5	29.3	13.3	7.8
Mahala and Sharma^61^	PSS	+	• PCM + fins • Gravels • PCM + fins + gravels	31.7 37.8 83.9	29.3 37.2 81.1	107 152 273	04.2 25.0 29.2	3.4 24.6 29.1
Present study	PSS	+	Clay pots facing					
			• upward (C-A2) • upward-downward (C-A3) • downward (C-A4)	48.1 55.3 64.0	49.8 55.9 65.3	10.2 21.6 36.3	30.9 33.9 37.5	30.9 34.3 37.9
			Black coated clay pots facing					
			• upward (C-A5) • upward-downward (C-A6) • downward (C-A7)	62.6 78.0 90.8	63.2 18.0 89.2	49.1 78.3 122.6	34.8 40.3 44.5	35.1 40.7 44.7

When considering configuration C-A7, there is a remarkable 90.8% increase in freshwater yield and a noteworthy 89.2% improvement in efficiency. Additionally, there is a substantial 44.5% reduction in CPL and a notable 44.7% decline in the payback period. These percentages surpass the outcomes reported in most other studies, as documented in Table 7, highlighting the effectiveness of the porous material in producing clean water from SS.

## Conclusions

In this study, the experiments were performed to evaluate the effects of porous material, both with and without black coating, and their arrangement on the effectiveness of SBPSS. This experimental investigation took place in Andhra Pradesh (16.6834°N, 80.3904°E), India, during March 2020. The key outcomes are sum up as follows:

- The SS equipped with porous material outperformed smooth SBPSS (C-A1).
- The water temperature experienced a significant enhancement when porous material was used in the solar still. Specifically, C-A4 exhibited an improvement of 11% in water temperature compared to C-A1. This improvement was further amplified to approximately 16% by applying black coating to the clay pots in configuration C-A7.
- Both clay pot arrangement and black coating play crucial roles in improving freshwater production in pyramidal-shaped solar stills. The combination of these factors leads to substantial yield gains, 48.09% for C-A2, 55.25% for C-A3, 64.01% for C-A4, 62.58% for C-A5, 78.03% for C-A6, and 90.76% for C-A7, making them effective strategies for enhancing solar still performance.
- Incorporating clay pots in the solar still system improves energy efficiency by 49.82% to 65.30%, depending on pot arrangement. Adding black coating intensifies the efficiency further (from 63.22% to 89.26%). Among the studied cases, the highest efficiency occurs when clay pots face downward (C-A4), reaching 28.10%. With black coating, this increases to 32.17% for C-A7.
- Including clay pots, both with and without black coating, in solar stills results in exergy efficiencies of 1.38% for C-A2, 1.52% for C-A3, 1.70% for C-A4, 1.86% for C-A5, 2.23% for C-A6, and 2.78% for C-A7, compared to the baseline efficiency of 1.25% for C-A1. The use of clay pots increases exergy efficiency by approximately 10.15% to 36.28% for solar stills (C-A2 to C-A4) when compared to C-A1. Additionally, coating clay pots with black material leads to substantial improvements over C-A1, with gains of 49.05% for C-A5, 78.27% for C-A6, and 122.58% for C-A7, highlighting the impact of black coatings on the exergetic sustainability of the solar still.
- The economic analysis reported that the addition of porous materials has significantly reduced the cost per liter (CPL) for different configurations. Specifically, C-A4 achieved a remarkable 37.5% drop in CPL compared to C-A1, with a CPL of Rs. 3.30. Additionally, applying a black coating to the porous materials further reduced CPL by about 44.5% for C-A7. This cost-effectiveness confirms the strategy’s productivity boost.
- Based on an average fresh water cost of Rs. 20 per liter, C-A4 generates an annual net profit of approximately Rs. 13,358, which increases to Rs. 15,598 for C-A7 with the black-coated clay pots.
- Using porous material in solar stills reduces payback periods compared to the smooth basin solar still (C-A1). Specifically, the payback periods are 246 days for C-A2, 234 days for C-A3, and 221 days for C-A4. Coating clay pots with black material further shortens payback periods: 231 days for C-A5, 211 days for C-A6, and 197 days for C-A7. The maximum reduction is about 37.9% for C-A4 and improves to about 44.7% for C-A7, making the investment more attractive.

The present study indicates that both black-coated and uncoated clay pots serve as cost-effective materials, contributing to sustainable development. Lower CPL and significant augmentation in distillate output, confirming the economic viability of the anticipated strategy. Furthermore, the payback period for solar stills with clay pots decreases significantly, and this reduction is even more pronounced for solar stills with black-coated clay pots, making the investment even more appealing. The ongoing research can be further expanded by incorporating cooling methods such as thermoelectric cooling, water and nanofluid glass cooling. These approaches have the potential to augment the condensation rate and eventually the distillate output of solar stills.

## Data Availability

The datasets used and/or analysed during the current study available from the corresponding author on reasonable request.
